# Progress and Prospects of Polymer-Based Drug Delivery Systems for Bone Tissue Regeneration

**DOI:** 10.3390/polym12122881

**Published:** 2020-12-01

**Authors:** Vyacheslav Ogay, Ellina A. Mun, Gulshakhar Kudaibergen, Murat Baidarbekov, Kuat Kassymbek, Zharylkasyn Zharkinbekov, Arman Saparov

**Affiliations:** 1 Stem Cell Laboratory, National Center for Biotechnology, Nur-Sultan 010000, Kazakhstan; ogay@biocenter.kz (V.O.); kudaibergen@biocenter.kz (G.K.); 2 School of Sciences and Humanities, Nazarbayev University, Nur-Sultan 010000, Kazakhstan; ellina.mun@nu.edu.kz; 3 Research Institute of Traumatology and Orthopedics, Nur-Sultan 010000, Kazakhstan; b.m.u.80@mail.ru; 4 Department of Medicine, School of Medicine, Nazarbayev University, Nur-Sultan 010000, Kazakhstan; kuat.kassymbek@nu.edu.kz (K.K.); zharylkasyn.zharkinbekov@nu.edu.kz (Z.Z.)

**Keywords:** polymer, nanoparticle, microparticle, drug delivery system, bone tissue regeneration

## Abstract

Despite the high regenerative capacity of bone tissue, there are some cases where bone repair is insufficient for a complete functional and structural recovery after damage. Current surgical techniques utilize natural and synthetic bone grafts for bone healing, as well as collagen sponges loaded with drugs. However, there are certain disadvantages associated with these techniques in clinical usage. To improve the therapeutic efficacy of bone tissue regeneration, a number of drug delivery systems based on biodegradable natural and synthetic polymers were developed and examined in in vitro and in vivo studies. Recent studies have demonstrated that biodegradable polymers play a key role in the development of innovative drug delivery systems and tissue engineered constructs, which improve the treatment and regeneration of damaged bone tissue. In this review, we discuss the most recent advances in the field of polymer-based drug delivery systems for the promotion of bone tissue regeneration and the physical-chemical modifications of polymers for controlled and sustained release of one or more drugs. In addition, special attention is given to recent developments on polymer nano- and microparticle-based drug delivery systems for bone regeneration.

## 1. Introduction

Bone tissue has a high regenerative ability, however, large bone defects produced by cancer, tumor resection, severe mechanical trauma, infectious diseases and congenital disorders do not recover spontaneously and require considerable amounts of bone grafting [1,2]. Insufficient regeneration of damaged bone tissue remains a common problem in orthopedics and traumatology. The standard surgical treatment for such defects is an autogeneic bone graft. Autografts possess major components for achieving osteoinduction, osteogenesis and osteoconduction after transplantation [3]. However, autologous bone grafting has a number of drawbacks related to a high risk of morbidity at the donor site, limited availability, prolonged wound drainage, large hematomas and reoperation [4]. In addition, the quality of the autograft can be highly variable depending on age and metabolic abnormalities of the patient [5]. To avoid these drawbacks and limitations, various bone substitutes such as allografts and xenografts are used in clinical practice [6]. Relative availability and absence of donor site morbidity make these bone substitutes a good alternative to the autograft. However, they have some disadvantages related to high risk of immunorejection and transmission of infections after transplantation, and possessing low osteogenic and osteoinductive properties [4]. Other commonly used bone repair techniques may involve distraction osteogenesis, bone cement fillers, pharmaceutical agents and soluble growth factors [5,6].

In repair and regeneration of damaged bone tissue, osteoinductive and angiogenic growth factors, such as bone morphogenetic proteins (BMPs), insulin-like growth factor (IGF), vascular endothelial growth factor (VEGF) and fibroblast growth factor (FGF) play an essential role [7]. BMPs are key factors in the reconstruction and restoration of damaged bone tissue [8]. BMPs have been shown to have powerful osteoinductive effects and the ability to stimulate the formation of new bone tissue due to the differentiation of mesenchymal stem cells (MSCs) into osteoblasts [9]. Some recombinant BMPs, such as BMP-2 and BMP-7, have been approved by the U.S. FDA and are currently being used in clinical practice to repair non-union fractures [10,11]. However, despite the high efficiency of recombinant BMPs, there are still some problems associated with their clinical use. This is primarily due to the short life of BMPs. Proteins loaded into the lesion site lose their biological activity in a short period of time, and therefore, large doses of recombinant BMPs are used in clinical practice in order to achieve complete bone regeneration [10]. For example, for BMP-2, an effective dose for bone regeneration is 1.5 mg/mL defect, which is 4–5 times higher than the endogenous dose. Such high doses of recombinant BMPs can diffuse from the lesion site and cause side effects, including abnormal bone overgrowth, osteolysis and an immune response [12]. 

In order to avoid these problems, there is a need to develop delivery systems that can help drugs safely mediate their biological activity for an extended period of time, protect from degradation, and control their slow release to the targeted site. To date, a number of natural and synthetic polymer-based systems have been designed and evaluated for the delivery of drugs for bone repair and regeneration [13,14]. Biodegradable synthetic polymers such as polyethylene glycol (PEG), poly(lactic-co-glycolic acid) (PLGA), poly(propylene fumarate) (PPF), polycaprolactone (PCL) can easily and readily be fabricated into desired shapes with relatively high mechanical strength, but they are not fully appropriate for the generation of bioactive and biocompatible tissues [15,16,17]. Natural polymers, such as collagen, gelatin, hyaluronic acid, fibrin, alginate and chitosan, are excellent scaffolds for cell growth and adhesion, but their weak mechanical properties render them susceptible to compression when transplanted into the defective area [18]. In this regard, approaches including extensive purification, crosslinking, or blending with synthetic polymers, have been used to improve the properties of natural polymers [19,20]. In this review, we discuss polymeric drug delivery systems of natural and synthetic origins for bone tissue regeneration. We also discuss the types of delivery systems and controlled release of growth factors and the potential use of polymer nanoparticle- and microparticle-based drug delivery systems in bone regeneration.

## 2. Types of Drug Delivery Systems

**Figure 1:**
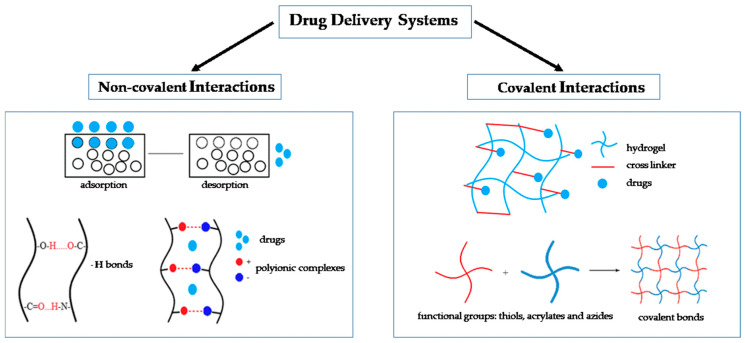
Types of drug delivery systems based on the method of interaction of a substance with a carrier-scaffold. Non-covalent interactions can be created through adsorption, H-bonds or polyionic complexes. Covalent interactions are the result of the formation of covalent bonds (between functional groups).

There are several approaches to classifying drug delivery systems (DDS). One approach involves dividing DDS into two main groups: uncontrolled and controlled. The first is characterized by the fact that the release rate of substances depends on the physical and chemical properties of the materials of the carrier [21]. For example, the release of substances from conventional polymer particles is usually controlled by a diffuse mechanism and degradation of the matrix. In controlled systems, the release of the substance can be regulated by interactions between the material of the carrier and its environment (pH, temperature, light intensity, etc.) Moreover, when changing the parameters of the structure of the carrier, it is possible to create a controlled delivery system of substances from an uncontrolled system [22,23,24,25]. The most common classification of DDS is their division based on the method of interaction of a substance with a carrier-scaffold (Figure 1). There are two main types of binding: non-covalent and covalent [26]. 

### 2.1. Non-Covalent Interactions

Non-covalent interactions of substances with a scaffold are often used to place biologically active substances and drugs on the carrier. Non-covalent interactions can be realized through passive adsorption of the substance on the surface of the scaffold, physical capture of the molecules of the substance in the scaffold-carrier through the formation of hydrogen bonds or ionic and hydrophobic interactions due to Van der Waals forces [27]. Adsorption of substances on the surface of the scaffolds is considered one of the most available methods for fabrication of DDS. For example, INFUSE^®^ and InductOS^®^ bone grafts are a simple mixture of recombinant human BMP-2 (rhBMP-2) and bovine Type I collagen, where rhBMP-2 is adsorbed onto the collagen matrix. A release of the substance loaded with adsorption method is mediated by desorption and biodegradation of the scaffold. The release kinetics of a drug has a high degree of dependence on properties of the carrier material and drug substance [28]. The main disadvantages of these DDS are insufficient control of the release kinetics and a low level of efficiency of their loading. Such delivery systems are characterized by explosive release, followed by the release of drugs, mediated by the process of biodegradation of the scaffold. The method for physical encapsulation or immobilization of drug substances in a scaffold is to mix biologically active substances and drugs with polymers prior to their gelation or hardening [29,30,31]. To create such scaffolds, natural and synthetic polymers such as collagen, fibrin, chitosan and polylactide are used. The main advantage of physical immobilization is that during the incorporation of the substance, no harsh chemicals are used in the scaffold, which reduces the risk of loss of activity of growth factors or drugs.

Ionic complexation is one option for non-covalent immobilization of substances in DDS. It is based on the creation of polyionic complexes from molecules of substances and functionalizing scaffolds, with different isoelectric points of the charged structure-forming molecules of the scaffold material. One example of this type of transport is the use of polyelectrolyte multilayer films, for example, as a carrier for recombinant BMP-2. The use of polyelectrolyte multilayer films with BMP-2 significantly increases the rate of bone healing [32]. However, in some cases, the formation of ionic complexes may be accompanied by a decrease in biological activity of the immobilized substance. A special case of non-covalent binding is affinity binding [27]. A number of polymers, such as fibrin, have specific biological sites with a high degree of affinity for certain regions of growth factors. Typically, DDS based on affinity binding have a more stable release profile compared to DDS with non-covalent binding. This activation method is physiological and today is positioned as one of the most suitable method for delivery of biologically active substances. For example, BMP-2 binds to heparin-conjugated fibrin and controlled delivery from fibrin hydrogels enhances their effect on formation and vascularization of a new bone [33].

### 2.2. Covalent Interactions

Covalent immobilization is a chemical interaction of biologically active substances with a matrix with the formation of covalent bonds (Figure 1). As a rule, for the implementation of this approach for the delivery of biologically active substances and drugs, it is important that the molecules contain reactive functional groups such as thiols, acrylates and azides [34,35]. Covalent binding of the substance with scaffold is not widely used, however this method of immobilization is required if the molecules of the loaded drug are not capable of non-covalent interaction with the material scaffold or non-covalent loading efficiency is extremely low [36]. It is known that this method can provide sustained drug release. Drug release kinetics in this case will be mediated by hydrolysis or cell-mediated enzymatic cleavage [37]. One positive property of covalent immobilization is control over the amount and the distribution of substances in the composition of the scaffold [14]. However, this approach has disadvantages associated with the complexity and the high cost of its preparation. Moreover, covalent immobilization can lead to conformational disturbances of drugs, which can significantly affect their biological activity [35]. Thus, a variety of DDS gives researchers a wide choice of approaches and methods for loading drug substances into scaffolds. At the same time, each method has its advantages and disadvantages. The development of improved approaches for drug delivery is a leading direction in the research and development of DDS. One possible option for optimizations is combining methods for loading substances into the carrier.

## 3. Biodegradable Polymers for Drug Delivery

To date, there are various biodegradable polymers being studied as DDS for bone tissue regeneration [14]. Biodegradable polymers are mostly applied where the transient existence of materials is required and they find applications as scaffolds for tissue regeneration, tissue adhesives and transient barriers for tissue adhesion, as well as DDS. Each of these applications demands materials with unique physical, chemical, biological and biomechanical properties to achieve efficient therapy. The polymers used for bone regeneration should be biodegradable, biocompatible, non-immunogenic and non-toxic to normal bone healing events. Biodegradable polymer materials fall into three general types. These are (1) natural polymers, of which collagen is the most widely used, (2) synthetic polymers, of which the poly(α-hydroxy acids) are most commonly used, and 3) a combination of these. Each type of polymer material has its advantages and drawbacks, which are summarized in Table 1.

### 3.1. Natural Polymers for Drug Delivery

Natural polymers have good biocompatibility and osteoinductive properties. In addition, they are safe, used in low concentrations and are capable of chemical modification and hydrogel formation. When used as grafts, these polymers are readily rearranged by the cells of the recipient. Moreover, the fibrous properties of polymers allow one to regulate the structure and porosity during the manufacture of scaffolds [18].

#### 3.1.1. Collagen 

Collagen is the most abundant protein in various tissues, including bone. Collagen scaffolds are very attractive for the creation of bioengineering structures since its mechanical properties can be changed by crosslinking with various chemical agents (glutaraldehyde, formaldehyde, etc.), or by their physical treatment (UV irradiation, heating, etc.) [38]. Collagen-based scaffold for drug delivery has frequently been used as a template to induce bone regeneration due to its safety and good biocompatibility. For the first time, bone regeneration was induced in femoral defects of rat and sheep and mandibular defects in dogs by BMPs in a collagen carrier in the early 1990s [39]. Several studies showed that recombinant human BMP-2, -4, -7 and -9 delivered with absorbable collagen sponge (ACS) effectively induced bone formation in a critically sized rat calvarial defect [40,41]. Currently, rhBMP-2 and rhBMP-7 absorbed on a collagen sponge are approved by the FDA for spinal fusion, tibial non-unions and oral-maxillofacial reconstructions [42]. However, clinical studies revealed a number of adverse side effects after transplantation of ACS loaded with high supraphysiological doses of rhBMP-2 or rhBMP-7 [43]. The strong burst release of high doses of BMPs from the collagen sponge in the first two days caused inflammation, swelling, ectopic bone formation and osteolysis in patients. In order to reduce these adverse events, differently modified collagen-based polymers for long-term delivery of rhBMP-2 were developed and evaluated, including an injectable collagen matrix, vitrified type I collagen membrane, apatite-coated collagen sponge and surface-treated collagen sponge [44]. For example, to enhance BMP-2 binding to collagen scaffolds, a porous collagen scaffold was chemically modified using Traut’s reagent and Sulfo-SMCC [45]. The modified collagen scaffold demonstrated a slower release of BMP-2 and preserved its biological activity. In addition, an in vivo study showed that subcutaneous implants of cross-linked collagen with immobilized BMP-2 led to more ectopic bone formation in experimental animals. 

Another study demonstrated that the controlled and sustained release of rhBMP-2 from collagen hydrogel can be regulated by blood plasma fibronectin [46]. Collagen hydrogel with fibronectin slowly released rhBMP-2 in the first 3 days which avoided adverse effects in clinics caused by the rapid release of large concentrations of rhBMP-2 from ACS. Recently, Lui and colleagues designed a mineralized collagen delivery system for BMP-2 and VEGF for promotion of bone formation and vascularization in mandibular defects. A mineralized collagen scaffold was prepared through self-assembling hydroxyapatite crystals that grew within the collagen fibrils to form triple collagen self-assembled helices. They showed that BMP-2 released from the mineralized collagen scaffold enhanced new mandibular bone formation. However, the combination of BMP-2 and VEGF exerted a synergistic effect and thereby significantly increased mandibular bone regeneration. It was suggested that the use of mineralized collagen delivery system for growth factors could provide a new therapeutic strategy to generate large-size vascularized bone grafts [47]. Despite the widespread use of ACS loaded with rhBMP-2 in clinical applications, recently it was found that rhBMP-9 is a more osteopromotive growth factor among the 14 BMPs [48]. It was revealed that ACS loaded with rhBMP-9, even at low concentrations of 10 ng/mL, was able to significantly induce over a two-fold increase in osteoblast differentiation and mineralization potential when compared to rhBMP-2 at a high concentration of 100 ng/mL. An in vivo study demonstrated that as little as 1 μg of rhBMP-9 loaded on ACS could promote new bone formation in a rat critically sized bone defect [41].

#### 3.1.2. Chitosan

Chitosan is a hydrophilic and positively charged linear polysaccharide prepared by alkaline deacetylation of crustacean chitin. The surface of chitosan is rich in amino functional groups that favor chemical or physical interaction with the nanoparticles, drugs, polymers and cells [49,50]. Despite a weak mechanical feature and a fast degradation rate, chitosan has many beneficial properties, such as its biocompatible, biodegradable, antibacterial, wound healing and bioadhesive properties that make it an excellent carrier for the safe and effective delivery of drugs and osteoprogenitor cells to sites for bone tissue regeneration [51,52].

Multiple studies demonstrated that chitosan scaffolds have a high potential as DDS to improve regeneration of damaged bone tissue [53,54]. However, to provide controlled and sustained release of osteopromotive drugs, certain chemical modifications in chitosan were proposed [51]. It was shown that photo-cross-linkable chitosan-lactide hydrogel with immobilized BMP-2 significantly enhanced osteoblast differentiation and mineralization of preosteoblast mouse bone marrow stromal cells and mouse myoblast cells due to the sustained release of BMP-2 from hydrogel [55]. In addition, the sustained release of BMP-2 from photo-cross-linkable chitosan-lactide hydrogel significantly enhanced mineralization in the cells. In another study, Kim and colleagues showed that photo-cross-linkable chitosan-lactide-fibrinogen hydrogels containing BMP-2 induced neo-osteogenesis and accelerated healing of the bone defects in a dose-dependent manner [56]. Several research groups developed a thiolated chitosan scaffold for effective BMP-2 delivery and bone formation [57,58]. For example, Bae and colleagues showed that thiolated chitosan was able to sustain delivery of BMP-2 and enhance osteoblastic differentiation of preosteoblast cells. Moreover, transplantation of thiolated chitosan for local delivery of BMP-2 induced more ectopic bone formation to a greater extent (1.8 fold) in comparison to collagen gel [57]. Heparinized chitosan scaffolds were also examined as potential carriers for BMP-2 delivery. It was shown that a heparinized chitosan-BMP-2 scaffold increased alkaline phosphatase (ALP) activity, calcium deposition and gene expression of osteocalcin and osteopontin in osteoblast cells compared to heparinized chitosan alone [59]. In another study, Kim and colleagues developed a novel heparinized methacrylated glycol chitosan (Hep-MeGC) carrier for demineralized bone matrix that can stabilize and enhance BMP-2 activity for increased osteoinduction [60]. Moreover, they demonstrated that Hep-MeGC hydrogels can effectively deliver bone marrow stromal cells, enhance endogenous activity of BMP-2 by sequestering and localizing the cell-produced BMPs and exert protective effects on bioactivity of BMP-2 against physiological stressors related to bone fracture healing.

#### 3.1.3. Hyaluronic Acid

Hyaluronic acid (HA) is a simple linear mucopolysaccharide containing repeating disaccharide units of N-acetyl-D-glucosamine and D-glucuronic acid [61]. HA has good biocompatibility, biodegradability, high viscoelasticity, hydrophilicity, non-immunogenicity, and can be easily modified and synthesized for bone tissue engineering applications [62,63]. HA is currently being used as a vehicle for drug delivery. Usually for controlled and sustained delivery of drugs or osteoinductive growth factors, chemically modified HA scaffolds are used. For example, Bhakta and colleagues demonstrated that in comparison to a collagen sponge, thiol-modified HA (Glycosil™) hydrogel exhibits a low burst followed by a sustained release of BMP-2 and leads to ectopic bone formation in experimental animals [64]. In another study, Kisiel and colleagues determined that incorporation of fibronectin (FN) fragment containing the major integrin-binding domain of full-length FN into HA hydrogel can improve attachment of MSCs and enhance the osteogenic potential of rhBMP-2 [65]. They also showed that delivery of rhBMP-2 with HA hydrogel containing FN resulted in significant ectopic bone formation with more homogeneous and organized collagen fibers compared to non-functionalized HA hydrogel with loaded rhBMP-2. Furthermore, 2-aminoethyl methacrylate conjugated HA hydrogel containing the growth and differentiation factor 5 and simvastatin was used for the stimulation of osteogenesis and repair of damaged parietal bone in rabbits [66,67]. An in vitro study demonstrated that hydrogel promoted osteogenic differentiation of adipose-derived stromal cells and increased expression levels of osteocalcin and osteopontin. However, an in vivo study showed that bone formation after implantation of methacrylate conjugated HA hydrogel was only slightly higher in comparison to control HA hydrogel. 

#### 3.1.4. Alginate

Alginate is a linear polysaccharide extracted from red and brown seaweeds and consists of 1,4-linked β-D mannuronic acid (M) and α-L-glucoronic acid (G) units. It is similar to chitin and chitosans in its chemical structure. In the presence of bivalent cations such as Mg^2+^, Ca^2+^, Ba^2+^ and Sr^2+^, these polysaccharides are exposed to ionotropic gel formation Alginate hydrogels have a good biodegradability, biocompatibility, non-immunogenicity and susceptibility for chemical modifications that make them suitable biopolymers for many biomedical as well as pharmaceutical applications [68]. Alginate is easily chemically modified due to a number of free hydroxyl (OH) and carboxyl (COOH) groups along its polysaccharidic structural backbone. To improve its properties, alginate hydrogel can be chemically modified by acetylation, phosphorylation, sulfation and esterification [69,70,71]. Nano-composite polymeric scaffolds (PLGA-PCL-nanoHA) containing sodium alginate hydrogel as the carrier for dual delivery of BMP-2 and basic FGF showed good sustained release ability, leading to significant promotion of the proliferation and osteogenic differentiation of MSCs [72]. In addition, an in vivo study revealed that the delivery of both growth factors can markedly enhance new bone formation and vascularization in large bone defect regeneration in rabbit mandibles compared with delivery of BMP-2 or basic FGF alone. 

In another study, Choi and colleagues fabricated core-shell microcapsules using poly(L-lactide-co-glycolide) (PLGA) and sodium alginate for dual delivery BMP-2 and dexamethasone (Dex) [73]. They observed an initial burst of Dex over 5 days, followed by a sustained release during 30 days, while the release kinetics of BMP-2 was more sustained. Moreover, it was found that released BMP-2 and Dex enhanced gene expression of ALP, osteocalcin and osteopontin in rat bone marrow stromal cells encapsulated with core-shell microcapsules into alginate hydrogels. The authors of the study suggested that dual controlled delivery of BMP-2 and Dex using core-shell microcapsules in alginate hydrogel can maintain the osteogenic potential of stromal stem cells. Kanczler and colleagues also demonstrated that alginate scaffold containing human bone marrow-derived MSCs, VEGF and BMP-2 was effective for regeneration of critically-sized femur defects in mice through the controlled and sustained release of growth factors [74]. Alginate hydrogel has also been applied as beads for multiple delivery of growth factors, for example for simultaneous delivery of BMP-2 and platelet rich plasma (PRP), which contains a number of growth factors such as FGF-2, platelet derived growth factor, tumor growth factor-β (TGF-β), IGF and VEGF. Fernandes and colleagues demonstrated that dual controlled release of PRP and BMP-2 from alginate beads significantly increased in vitro proliferation, osteogenic differentiation and mineralization activity of MSCs [75].

#### 3.1.5. Fibrin 

Fibrin is a natural biopolymer endowed with adhesive, sealant and hemostatic properties, and is suitable for cell adhesion and tissue stability [76]. Fibrin is widely used as scaffolds and DDS, enabling the precise application of biomaterials, cells, growth factors and nanoparticles [77]. Traditional commercial fibrin hydrogels have been produced from thrombin and fibrinogen of human plasma in the presence of calcium ions [78]. Fibrin has multiple binding sites for cells, growth factors and extracellular matrix (ECM) components. Thus, fibrin can provide main growth factors required for bone regeneration including BMP-2, TGF-β, FGF, VEGF, PDGF and IGF [79]. However, in other cases, the covalent binding of these growth factors to fibrinogen is considered. It generally requires certain chemical modifications with reactive functional groups allowing a stable association [37]. For example, to fabricate an injectable fibrin hydrogel for sustained delivery of BMP-2, Yang and colleagues synthesized heparin-conjugated fibrinogen with a carbodiimide chemistry method to keep BMP-2 for a long duration of time [33]. They showed that BMP-2-loaded heparin-conjugated fibrin (HCF) hydrogel controlled and sustained release of BMP-2 for 13 days in comparison to fibrin hydrogel. BMP-2 released from HCF significantly increased ALP activity in cultured osteoblasts and significantly enhanced ectopic bone formation in rats. Moreover, a comparative study demonstrated that BMP-2-loaded HCF hydrogel is more effective for stimulation of bone regeneration than BMP-2-loaded collagen sponge [80]. 

Recently, Sanchez-Casanova and colleagues developed a novel technological platform for spatiotemporal control of BMP-2 production using a heat-activated and dimerizer-dependent transgene expression system that was incorporated into MSCs [81]. They encapsulated genetically engineered MSCs in hydrogels based on fibrin and plasmonic gold nanoparticles that transduced incident energy of an NIR laser into heat. In the presence of a dimerizer, photoinduced mild hyperthermia induced the release of bioactive BMP-2 from NIR responsive cell constructs. An in vivo study showed that induction of NIR-responsive cell constructs conditionally expressing BMP-2 in a critically sized bone defect bone defect resulted in the formation of new mineralized tissue in mice.

### 3.2. Synthetic Polymers for Drug Delivery

In tissue engineering and drug delivery, synthetic polymers are used as carriers for peptides, drugs, genes, proteins, etc. Synthetic polymers are biocompatible and decompose into non-toxic products [82]. The most commonly used synthetic polymers in tissue engineering are poly (lactic acid) (PLA), poly(lactic-co-glycolic acid) (PLGA), poly (ε-caprolactone) (PCL) and polyethylene glycol (PEG) [83]. 

#### 3.2.1. Poly (ε-caprolactone)

Poly (ε-caprolactone) (PCL)—elastic, biocompatible, bio absorbable and biodegradable linear aliphatic polyester, has good environmental stability, is easy to process and is used in the development of biomaterials. PCL is a semicrystalline polyester, with a melting temperature around 55–60 °C and a Tg of 60 °C, synthesized by the ring-opening polymerization of ε-caprolactone. PCL is soluble in a wide range of organic solvents and is easily processed into different structures [84,85]. However, as known, pure PCL polymer is not suitable for use in bone regeneration due to its mechanical properties and degradation. In addition, PCL, like many synthetic polymers, is hydrophobic, which limits its polymer–cell interaction [86,87]. Generally, PCL is used as one of the material components [88,89], so minerals such as hydroxyapatite (HA), tetracalcium phosphate (TCP), etc. [90,91,92] are added to the composites to improve the mechanical strength of PCL scaffolds. Xiaogang Bao and colleagues obtained a “functional artificial bone” based on a 3D printing scaffold from HA and PCL. Implanted scaffolds in rabbit tibia defect (1.2 cm) showed better defect repair results in 12 weeks, similar to autogenous bone graft, compared to the original gel/scaffold. The cytokine-loaded hydrogel has good biodegradability and can stably release VEGF-165/BMP-2 [93]. Yao Q.Q. and colleagues succeeded in synthesizing microspheres-aggregated 3D PCL scaffolds of various structures (macro/micro/nano). The scaffold provided a significantly slow release of the low molecular weight drug (phenamil) and BMP-2 [94]. By obtaining bilayer scaffolds by sequential electrospinning of a PCL solution containing a decellularized (dECM), Junka R. and colleagues demonstrated that the incorporation of dECM into polymer fibers limits the access of phenotypes that disrupt bone formation during the healing process [95]. In another study, Yao Q. and colleagues fabricated 3D electrospun PCL/PLA blend (mass ratio: 4/1) nanofibrous scaffolds with high porosity (~96%). PCL/PLA scaffolds improved osteogenic differentiation of human MSCs, showed homogeneous scaffold cellularity and increased cell viability, osteogenic gene expression and apatite-like deposition in vitro. The results of new bone formation in a model of a skull bone defect in mice of critical size in vivo showed that with the same amount of rhBMP2 (0.75 μg) added per scaffold group, the greatest bone formation was observed in the PCL/PLA-rhBMP2 group (4.56%) than in the PCL-rhBMP2 group (0.99%) [96].

#### 3.2.2. Poly(lactic-co-glycolic acid)

Poly(lactic-co-glycolic acid) (PLGA) is also a biocompatible synthetic copolymer of poly-L-lactic acid (PLLA) and polyglycolic acid (PGA), which is used in medical applications, therapeutic tools and DDS. PLGA is synthesized by ring-opening copolymerization of two different monomers of glycolic acid and lactic acid [97,98,99]. Cao S.S. and colleagues used two relatively distinct 3D printing (3DP) technologies to produce 3D poly(lactic-co-glycolic acid)/β-tricalcium phosphate (3D-PLGA/TCP) and 3D beta-tricalcium phosphate (3D-TCP) scaffolds. Administration of TCP increased the adhesion and proliferation of human dental pulp stem cells (hDPSC) [100]. Another group of scientists used a cryogenic 3D printing method to develop a bioactive scaffold of PLGA/β-TCP, in which graphene oxide (GO) and BMP-2 were loaded in situ (PTG/P). The pore size averaged 400 ± 50 μm. Rat MSCs were cultured directly on a scaffold. In an in vivo study, the authors successfully corrected the rat cranial bone defect [101]. Deng and colleagues using a synthesized scaffold based on 3D-printed PLGA/HA/chitosan/rhBMP-2, were able to successfully repair a bone defect in vivo in the mandible of a rabbit model. In an in vitro experiment, a gradual release of rhBMP-2 from scaffold was observed [102]. There are also known studies using bone morphogenetic proteins BMP-6 and BMP-7, which, when combined with scaffold, can promote bone regeneration in vivo [103,104]. Jakus, A.E. and others were able to 3D print hyperelastic “bone” based on 90 wt% hydroxyapatite and 10 wt% polycaprolactone or poly(lactic-co-glycolic acid). In in vitro studies with human MSCs derived from bone marrow, the scaffolds showed good cell viability and proliferation and induced osteogenic differentiation after four weeks without any osteo-inducing factors in the medium. The authors conducted in vivo studies on a mouse subcutaneous implant model for biocompatibility (7 and 35 days), on a posterolateral fusion in rats for new bone formation (8 weeks) and in a non-human primate calvarial defect case study (4 weeks). Thus, they demonstrated the efficacy of hyperelastic “bone”, which did not induce a negative immune response, became vascularized, quickly integrated with surrounding tissues, quickly ossified and supported the growth of new bone without using the biological factors [105].

#### 3.2.3. Poly(lactic acid)

Poly (lactic acid) (PLA) is a bioresorbable material and exists in three forms—D-PLA, PDLA and L-PLA (PLLA). The polymerization of these monomers leads to the formation of semicrystalline polymers and the degree of crystallinity depends on the molecular weight and polymer processing parameters. This polymer can be degraded into nontoxic components with a controllable in-vivo degradation rate and has a long history of use in DDS and tissue engineering scaffolds [106,107]. PLA-based biopolymers are often synthesized in various modifications [98,108,109,110]. Zhang H. and colleagues obtained a PLA/HA 3D printing scaffold that was cultured with bone marrow stromal cells embedded in the vascular bundle and surrounded by a periosteal capsule and examined after four and eight weeks. PLA/HA scaffold had better osteogenic capacity and osteoinductive activity, and created an environment for bone development [111]. Mohammadi M. and colleagues synthesized electrospun PLA nanofibers to stimulate osteogenic differentiation of MSCs. The resulting scaffolds provided a sustained release profile of BMP-2 peptide up to day 21, while maintaining cell attachment and proliferation without cytotoxicity [112]. D. Ma and colleagues used PLA/PLGA polymeric particles as vehicles for rhBMP-2 transport. Particles of PLA/PLGA suppressed the pulsed release characteristics of BMP-2 [113]. Other researchers have synthesized hierarchical macroporous biocompatible (HmPB) scaffolds based on PLLA and PCL, which are readily fabricated by solvent evaporation of 3D printed Pickering HIPEs stabilized by h-SiO_2_ nanoparticles. In vitro studies show that this HmPB is a suitable drug carrier. Loaded 1.2 wt.% enrofloxacin released almost 80% in the first 2.5 h, and the release rate of all samples reached more than 98% after 10 h. The resulting scaffold is structurally similar to the natural ECM, thereby promoting good biocompatibility and cell adhesion of mouse bone marrow-derived MSCs [114]. In another study, Ren and colleagues synthesized nano-fibrous meshes of poly (L-lactide) (PLLA) and gelatin (1:1 in weight ratio). The study showed that the use of nano-fibrous meshes promoted osteogenic differentiation of bone marrow-derived MSCs compared to the control. The resulting composites were implanted into rat skull defects. As shown by the results in comparison with the control group, 12 weeks after surgery in the groups filled with composite, there was a significant formation of new calcified bone [115].

#### 3.2.4. Polyethylene Glycol

Polyethylene glycol (PEG) is a hydrophilic non-ionic polymer, non-toxic and non-immunogenic, soluble in aqueous solutions and organic solvents [116,117,118]. PEG is suitable for making composites with other polymers. So to improve its biocompatibility and degradation rate, Sinan Eğri and colleagues fabricated scaffold PLA-PEG-PLA terpolymers. The release of BMP-2 from the synthesized scaffold was much slower (by several months) than that of VEGF (2 days after insertion). The addition of BMP-2 and VEGF to scaffold PLA-PEG-PLA led to an increase in cell adhesion and proliferation [119]. Lin Dan and colleagues performed work on the reconstruction of bone-cartilage defects using PEGylated poly (glycerol sebacate) (PEGS) with a macro-/microporous surface. PEGS served as one of the layers of a two-layer scaffold PEGS/mesoporous bioactive glass (MBG). As shown by in vivo results for 12 weeks, the two-layer scaffold viscoelastic PEGS/MBG successfully reconstructed cartilage and subchondral bone, demonstrating exceptional regenerative efficiency [120]. In another study, Yang Y. and colleagues fabricated a novel class of bone-targeting anabolic compound based on PTH-PEG-BP (parathyroid hormone- polyethylene glycol-bisphosphonate) for the treatment of osteoporosis and related bone disorders. According to the authors, in vitro and in vivo studies showed that PTH-PEG-BP conjugates had significant enhanced affinity for bone mineral and improved the trabecular bone volume and structural strength [121].

## 4. Polymer Nanoparticle- and Microparticle-Based Drug Delivery Systems for Bone Regeneration

There are four key aspects that the bone tissue engineering field is aiming for in providing a successful bone regeneration at the defect site with minimal complications: (1) osteogenic cells that generate bone tissue matrix; (2) a biocompatible framework (carrier) that mimics the natural ECM of the bone; (3) vascularization; (4) morphogenetic signals that direct the cells [122]. The general idea behind bone tissue engineering is that eventually the bioactive framework should be resorbed and replaced by the newly regenerated tissue of the body. Therefore, the biomaterial should be both osteoinductive and osteoconductive, as well as capable of integrating into surrounding bone [98]. Additionally, the biomaterial should be feasible in preparation and sterilization and possess stable mechanical and chemical properties in the host environment and non-thrombogenic activity [122]. Polymer nanoparticles are a promising class of biomaterials that meet these criteria. Nanoparticle-based DDS have been attracting considerable attention in biomedical and pharmaceutical sciences over the last two decades due to their unique physicochemical properties, including their ultra small size, high reactivity and large surface area to mass ratio, which can offer significant benefits compared to traditional therapeutic and diagnostic agents [123]. They have been successfully applied as drug carriers [124,125], diagnostic tools [126,127], labelling and tracking agents [128,129]. Among various types of nanoparticles, polymer nanoparticles are of particular interest for drug delivery as they exhibit certain advantages over their counterparts, which include controllable size and size distribution, longer clearance time, lower toxicity, higher drug loading capacity and good biocompatibility [130]. 

Chitosan is one of the most abundant natural amino polysaccharides. Chitosan-based nanoparticle DDS are very promising in biomedical fields and tissue engineering, due to their non-toxic, biocompatible, biodegradable, non-carcinogenic and antibacterial properties [131,132]. However, chitosan exhibits low mechanical characteristics and non-osteogenic inductivity, hence, has limited functions in bone tissue engineering [131,133]. To overcome this limitation, hybridized nanocomposite formulations of chitosan and other natural or inorganic components are often used for adequate application in tissue engineering. Nanocomposite formulations in tissue engineering are aiming to combine the mechanical properties of inorganic moiety and improved degradation and compatibility profiles of a polymer component [134]. Incorporation of TiO_2_ nanoparticles into chitosan sponge resulted in the formation of novel scaffold for bone tissue engineering with improved bone regenerating capability [135]. Even distribution of TiO_2_ nanoparticles on the surface of chitosan sponges provided stronger mechanical properties of the composite, while biological activity also demonstrated promising results. Nano chitosan–TiO_2_ (50%) scaffolds were capable of osteogenesis, showing the highest expression on Dentin Matrix Protein 1 and osteocalcin gene. Ikono and colleagues demonstrated that chitosan-TiO_2_ nanocomposites exhibited improved robustness, biomineralization, osteogenic induction and biocompatibility, which makes them attractive in the bone tissue engineering field, particularly in cases with complex defects and cavities [135]. Another chitosan-based nanocomposite modified with TiO_2_ was later reported by another group to mimic the human bone ECM. A polymer film-construct of Chitosan/poly (vinyl alcohol)/Nanohydroxyapatite (CPHT I-III) doped with TiO_2_ nanoparticles demonstrated enhanced mechanical and biological properties, highly cytocompatible nature for osteoblast-like MG-63 cell attachment and proliferation [136]. Various inorganic components could be applied to the formation of chitosan-hybrid nanocomposites, such as silica [137], POSS (the smallest silica particle) [138], bioglass [139], as well as polymers [140,141], resulting in improved mechanical properties, antimicrobial activity, cytocompatibility, osteoblast adhesion, osteocalcin secretion and biomineralization of cells, thus providing a promising biomaterial for bone tissue engineering. 

Other biological polymers, such as hyaluronic acid, gelatin, alginate and collagen, can also be used for nanocomposite fabrication for bone tissue regeneration [142,143,144,145,146]. Purohit et al. reported the successful formation of gelatin-alginate nanocomposite scaffold with incorporation of cerium oxide nanoparticles (nanoceria). The effect of nanoceria concentration on mechanical and biological properties of nanocomposite was evaluated, demonstrating improved bio-mineralization properties and decreased in vitro weight loss of a scaffold. Gelatin-alginate-nanoceria composites exhibited a high potential to accelerate differentiation of MSCs to osteoblast and reduce free radicals [147]. Hyaluronic acid was shown to optimize a nanoparticle formulation for delivery of alendronate (a bone anti-resorptive agent) and curcumin (a bone density enhancing drug) inducing an increase in proliferation, differentiation and mineralization of MC3T3-E1 cells leading to improved bone formation [148]. 

Along with natural polymers, synthetic polymers also demonstrated promise as candidates in the bone tissue engineering field owing to reproducible physical and mechanical properties, biocompatibility and adjustable biodegradation kinetics [149]. The most often used synthetic polymers for bone regeneration include poly(lactic acid) (PLA), poly(glycolic acid) (PGA), poly(lactic acid-co-glycolic acid) (PLGA) and poly(-caprolactone) (PCL). The table below summarizes the most recent and relevant publications on both natural and synthetic polymer-based nano- and micro-formulations for bone tissue engineering (Table 2). Synthetic polymers have found their wide application in the development of 3D-printed materials for bone regeneration [150,151,152]. Polycaprolactone-based nanocomposite 3D matrix with inorganic nanoparticles incorporated were shown to significantly enhance osteogenic differentiation in vitro and to exhibit tissue mineralization and early bone defect repair [153]. Polycaprolactone nanocomposites also demonstrated better cell attachment, proliferation and enhanced calcium phosphate deposition, suggesting a high potential as bone tissue engineering material [154]. Similarly to natural polymers, synthetic polymers significantly improve proliferation, cell attachment and anchorage, however, they are low in osteopromotive properties, hence cannot provide complete bone tissue regeneration alone [155]. A mineral-based component is crucial in tissue restoration, therefore minerals are used for nanoscaffold fabrication for bone regeneration, for example hydroxyapatite (HA) [156]. Often, natural and synthetic polymers are employed simultaneously in the development of nanocomposites for bone repair. A nanocomposite of PLGA-HA-chitosan loaded with BMP2 provided a sustained release of the growth factor, induced osteogenic effects and demonstrated a successful bone defect repair in vivo [102]. Another three-layered composite membrane based on PLGA and collagen was reported to be a promising platform for guided tissue regeneration under the concept of functional graded material [157]. 

Although nanosized materials are currently recognized to be more bioactive than their microsized counterparts, there is a series of recent studies demonstrating the high potential of PLGA microparticles in bone regeneration. The performance profiles of PLGA micro- and nanoparticles loaded with simvastatin, a cholesterol lowering drug, were compared and their effects on bone regeneration were studied in vitro and in vivo [158]. PLGA microspheres exhibited a sustained release of simvastatin for one month, as opposed to one week for PLGA nanospheres. Simvastatin-loaded PLGA microspheres were also reported to induce the proliferation of MC3T3-E1 cells and increased ALP activity. PLGA nanospheres, on the other hand, did not show such properties [159]. These data suggested that PLGA microparticles exhibit far superior properties, compared to PLGA nanoparticles, in bone tissue regeneration. An earlier study also reported that PLGA microparticles loaded with simvastatin demonstrated their high potential for bone regeneration [160]. PLGA microspheres could serve as excellent platforms for the formation of DDS with a high promise for bone tissue engineering. PLGA-alginate core-shell microspheres provided a controlled release of magnesium ions that exhibited osteogenic tendency in vitro. This PLGA-based delivery system stimulated in situ bone regeneration in vivo, enhanced bone mineral density [161]. Injectable PLGA microspheres with tunable magnesium ion release promoted attachment, proliferation, osteogenic differentiation and, particularly, cell migration of bone marrow-derived mesenchymal stromal cells [162]. PLGA microparticles were reported to be effective in treating infections and bone defects caused by *Staphylococcus aureus*. PLGA microparticles were assembled with human demineralized bone matrix in the development of antimicrobial bone paste with a controlled release of vancomycin hydrochloride. This DDS provided a sustained release of antibiotic for over two weeks, effectively inhibiting growth of the bacteria, making the PLGA paste promising for the controlled delivery of bioactive molecules for bone repair. 

The future of polymer nano- and microparticle formulations for bone tissue regeneration is undoubtedly highly promising and merits further investigation. Over the past few decades, tremendous progress has been made in the development of nanocomposites and micro-formulations employing both natural and synthetic polymers, studying their characteristics and eventually contributing to the field of tissue engineering. The novel nano- and micro-formulated biomaterials are designed for the delivery of drugs, cells and growth factors, promoting antimicrobial activity, biomineralization, improving mechanical and biological properties of the platform, and hence, bone tissue repair and formation. However, further understanding of the interaction at the cell/surface interface, as well as a closer look at the toxicity, hemotoxicity and long-term biocompatibility of the material and the host tissue are required for clinical applications. This overall highlights the importance of close interaction and inter-disciplinary collaboration of medical, biological, chemical and material communities to move from the bench to clinical applications.

## 5. Clinical Trials of Polymer-Based Drug Delivery Systems for Bone Tissue Regeneration

Despite a number of developed polymer-based DDS to treat bone defects, only a few have reached clinical application. Currently, there are several commercially available carriers for delivery of osteoinductive growth factors such as OP-1, INFUSE^®^, InductOS^®^ and AUGMENT^®^ [8]. OP-1 was the first FDA approved osteoinductive biomaterial containing rhBMP-7, type I bovine collagen matrix and the putty additive carboxymethyl cellulose sodium [163,164]. A number of prospective, randomized, multicenter studies have demonstrated that OP-1 is safe and very effective for the treatment of open tibial fractures, distal tibial fractures, tibial nonunions, scaphoid nonunions, atrophic long bone nonunions and the posterolateral spine [165,166]. For example, Ristiniemi and colleagues investigated the efficacy of OP-1 in the treatment of distal tibial fractures in a case-control study in 40 patients [166]. They divided the patients into two groups: OP-1 (20) and without OP-1 (20). The results of this clinical study showed that implantation of OP-1 significantly accelerated bone regeneration in distal tibial fractures compared to the control group (6.3 months vs. 9.0 months). In clinical practice, INFUSE^®^ Bone Graft is widely used for the treatment of bone fractures, nonunions, interbody spinal fusions and in oral maxillofacial surgery [167]. It is the most studied osteoinductive biomaterial and is one of the most significant advances in traumatology and orthopaedics. INFUSE^®^ Bone Graft consists of rhBMP-2 (1.5 mg/mL) and ACS as a carrier. Multiple clinical trials conducted from 2002 to 2017 to evaluate the safety, efficacy and dose-dependent effects of INFUSE^®^ implantation in anterior cervical discectomy and fusion (ACDF) demonstrated that patients treated with rhBMP-2/ACS had significantly higher fusion rates compared to non-rhBMP-2 patients, not only in total value but also in 3 tiers of rhBMP-2 doses [168]. It should be noted that ACS loaded with a low dose of rhBMP-2 (<0.7 mg/level) showed the highest fusion rate among all rhBMP-2 doses. However, patients treated with rhBMP-2/ACS had a higher complication rate, dysphagia and wound infections than that in the non-rhBMP-2 group. In 2-level ACDF, the fusion rate was significantly higher in rhBMP-2 treated patients than non-rhBMP-2, but not in the complication rate.

Furthermore, a randomized clinical trial (RCT) with 160 patients demonstrated the efficacy of rhBMP-2/ACS in comparison with commonly used autogenous bone graft for maxillary sinus augmentation [169]. The bone density was higher in the rhBMP-2/ACS group and the success rate of implant integration reached 79% (exceeding the targeted success rate of the protocol by 6%). Additionally, application of rhBMP-2/ACS helped avoid the long-term presence of complications such as prolonged paresthesia, gait disturbance and pain, which are commonly associated with the graft harvesting procedure. Moreover, rhBMP-2/ACS can be effective in bone synthesis for the placement of dental implants. RCT on 80 patients demonstrated that delivery of 2 doses (1.5 mg/mL) of rhBMP-2/ACS after tooth extraction increased bone augmentation in the alveolar ridge, and that the adequacy of bone formation was at least twice greater in the rhBMP-2/ACS group than in the placebo or no treatment groups [170]. However, another RCT with 177 patients with open tibial fractions demonstrated no significant acceleration of bone synthesis in comparison with routine soft-tissue management [171]. Proportion of fracture healing was higher by 12% in the rhBMP-2/ACS group at week 13, however, it demonstrated almost the same results at week 20 (68% for the rhBMP-2/ACS group and 67% for the control group). Furthermore, a recent systematic review of polymer-based delivery of BMP-2 in 17 RCTs for the treatment of craniofacial bone defects did not find evidence of the efficacy rhBMP-2/ACS and rhBMP/hydrogels with HA in the maxillary sinus augmentation and the reconstruction of cranial vault defects. At the same time, the authors state that the treatment with rhBMP-2/ACS can be superior for alveolar ridge augmentation, alveolar cleft reconstruction and in spine surgery. More clinical trials are needed to evaluate the efficacy of rhBMP-2/ACS and rhBMP/hydrogels treatments for the reconstruction of mandibular bone and osteodistraction [10]. 

More recently, Min and colleagues reported on the administration of rhBMP-2/ACS for medication-related osteonecrosis of the jaw (MRONJ) treatment [172]. They demonstrated that rhBMP-2/ACS (0.5 mg/mL) could promote bone healing by enhancing bone remodeling in MRONJ patients more than 6 months after surgery. Another research group used a higher dose of 1.5 mg/mL of rhBMP-2 to treat MRONJ patients [173]. They reported that large critical-sized defects of the mandible in MRONJ patients were completely repaired in all 3 patients in 3 months after implantation of rhBMP-2/ACS.

Like BMPs, platelet-derived growth factor (PDGF) also plays an important role in bone regeneration [174]. Several recent clinical studies reported that β-TCP-collagen loaded with rhPDGF-BB (Augment^®^ Injectable Bone Graft, Wright Medical Technologies) is effective in ankle and hindfoot fusions [175,176]. Prospective RCT showed that rhPDGF-BB/β-TCP-collagen significantly reduced the time needed for complete fusion (14.3 ± 8.9 weeks) in comparison to autograft-treated patients (19.7 ± 11.5 weeks). Moreover, clinical success rate was achieved in 57 of 63 (91%) rhPDGF-BB/β-TCP-collagen-treated patients and 120 of 154 (78%) autograft-treated patients at 52 weeks [175]. The results of another RCT of rhPDGF-BB/β-TCP-collagen with autograft in ankle or hindfoot arthrodesis using a propensity score (PS) subclassification cohort study design showed that rhPDGF-BB/β-TCP-collagen rhPDGF-BB/β-TCP-collagen was as effective as an autograft for ankle and hindfoot fusions, with less pain and morbidity than treatment with autograft [176]. Thus, commercially available polymer-based DDS for bone regeneration demonstrated good therapeutic efficacy in various clinical applications.

## 6. Conclusions

Multiple studies showed that biodegradable natural and synthetic polymers play a key role in the development of innovative drug delivery systems and tissue engineered constructs, which improve the treatment and regeneration of damaged bone tissue. Several types of biodegradable and biocompatible polymers have been studied as potential drug delivery systems, including nano- and microparticles. Polymers after chemical or physical modifications acquire improved properties for controlled and sustained release of various drugs, including osteoinductive growth factors for bone regeneration. A special place among the various approaches for drug delivery is occupied by scaffolds with incorporated nano- and microparticles loaded with drugs or biological active substances. These innovative drug delivery systems have a number of advantages that distinguish them from other systems. The small size and high specific surface of nano- and micro-particles provide high efficiency for loading several drugs simultaneously. Their composition and physico-chemical properties allow for the protection of the loaded drugs from enzymatic digestion and other aggressive agents in the wound environment. Moreover, use of nano- and microparticles allows for more effective and controlled drug release from scaffold over time in relevant therapeutic concentrations. These controlled drug delivery systems are able to effectively stimulate osteogenesis and accelerate bone regeneration without significant side effects. Despite the promising results in preclinical studies, implementation of additional clinical trials is required to enhance the translation of developed innovative drug delivery systems for effective bone healing.

## Figures and Tables

**Table 1:** Advantages and limitations of polymer-based drug delivery systems for bone tissue regeneration based on a polymer type.

Source	Polymer	Advantages	Limitations	Reference
Natural	Collagen	The most abundant protein, capable of altering its mechanical properties by crosslinking, safety, biocompatibility	Strong burst release, causing inflammation, swelling, ectopic bone formation and osteolysis	Busra and Lokanathan, 2019 Sheikh et al., 2015 Zara et al., 2011
	Chitosan	Biocompatibility, biodegradability, antibacterial and wound healing activity, bioadhesion, presence of functional groups on the surface, enabling physical and chemical functionalization	Requirement for additional modifications for controlled and sustained release of osteopromotive drugs	Levengood and Zhang, 2014 Saravanan et al., 2016 Venkatesan et al., 2017
	Hyaluronic acid	Good biocompatibility, biodegradability, high viscoelasticity, hydrophilicity, non-immunogenicity, capability to modification, low burst release, sustained drug release	Low mechanical and physical properties, fast degradation rate, complicated crosslinking method	Hemshekhar et al., 2016 Zhao et al., 2016, Kim et al., 2019
	Alginate	Good biodegradability, biocompatibility, non-immunogenicity, capability of modification, significant promotion of the proliferation and osteogenic differentiation	Poor cell attachment ability, limited availability of cellular adhesion sites	Ray et al., 2020 Su et al., 2013, Purohit et al., 2020
	Fibrin	Adhesion, hemostasis, sealant capability, presence of multiple binding sites	Requirement for additional modification	Bujoli et al., 2019 Noori et al., 2017 Khodakaram-Tafti et al., 2017 Martino and Hubbell, 2010 Censi et al., 2012
Synthetic	Poly (ε-caprolactone)	Elasticity, biocompatibility, bioabsorbtion and biodegradability	Limited polymer-cell interaction due to its hydrophobicity and mechanical properties, used as one of the scaffold components	Narayanan et al., 2016 Ghassemi et al., 2018 Miroshnichenko et al., 2019 Aldemir Dikici et al., 2019 Pazarçeviren et al., 2020 Wang et al., 2017
	Poly(lactic-co-glycolic acid)	Biocompatibility, biodegradability, increased adhesion and cell viability and proliferation, sustained release of GF, induced osteogenic differentiation, quick integration with surrounding tissues	Rapid biodegradation	Turnbull et al., 2018 Bharadwaz and Jayasuriya, 2020 Cao et al., 2020 Zhang et al., 2019 Deng et al., 2019 Das et al., 2016 Kim et al., 2019 Jakus et al., 2016 Generali et al., 2017
	Polyethylene glycol	Hydrophilicity, biocompatibility, non-immunogenicity, biodegradability	Absence of functional groups on the surface, used as one of the composite components	Saroia et al., 2018 Wang et al., 2019 Kutikov and Song, 2015 Eğri and Eczacıoğlu, 2017

**Table 2:** A summary of the most recent and relevant publications on nano- and micro-particle polymer formulations for bone regeneration.

Source	Polymer	Formulation	Function	Reference
Natural	Chitosan	Nanocomposites	Improved osteoblast adhesion and proliferation, osteocalcin secretion and biomineralization of cells	Tamburaci et al., 2020
		Sponge	Improved biomineralization and osteogenic induction	Ikono et al., 2019
		Nanocomposite film	Accelerated tissue ingrowth, enhanced capability to mimic human bone extracellular matrix, vascularization, antibacterial efficacy, compatibility with human erythrocytes, advanced cell attachment and high proliferation with human osteoblasts	Khan et al., 2019
		Nanoparticle-containing electrospun fibers	Increased bioavailability and osteogenic capability	Balagangadharan et al., 2019
		Coated nanoparticles	Improved antimicrobial activity	Ignjatović et al., 2016
	Hyaluronic acid	Nanocomposite	Improved antibacterial activity, high bone differentiation of mesenchymal stem cells	Makvandi et al., 2020
		Nanoparticle	Sustained delivery of alendronate and curcumin, increased proliferation, differentiation and mineralization of MC3T3-E1 cells	Dong et al., 2018
	Gelatin	Composite scaffold	Improved cell proliferation and bone healing, cytocompatibility, osteoinductivity	Hashemi et al., 2020
		Nanocomposite scaffold	Accelerated differentiation of mesenchymal stem cells to osteoblast and reduce free radicals	Purohit et al., 2020
		Nanotubes/hydrogel	Enhanced osteogenic differentiation, osteoimmunomodulatory and antibacterial activities	Ou et al., 2020
	Alginate	Nanocomposite scaffold	Improved bio-mineralization	Purohit et al., 2020
		Microbeads	Enhanced bone formation, higher injectability and washout resistance	Amirian et al., 2020
	Collagen	Nanocomposite	Enhanced bone regeneration, improved healing and tissue remodeling	Patel et al., 2020
		Three-layered composite membrane	Guided tissue regeneration	Liao et al., 2005
Synthetic	Poly(-caprolactone) (PCL)	Composite nanofibers	Improved fiber morphology, cell attachment, proliferation, differentiation, biomineralization, calcium-phosphate deposition	Awasthi et al., 2020
		Nanocomposite 3D matrix	Enhanced osteogenic differentiation, early bone defect repair, tissue mineralization	Shen et al., 2019
	PLGA	Nanocomposite	Induced osteogenic effects and successful bone defect repair in vivo, BMP2 delivery	Deng et al., 2019
		Microspheres	Controlled delivery of magnesium ions, enhanced cell attachment, proliferation, osteogenic differentiation, cell migration of bone marrow mesenchymal stromal cells, promotion of mineral depositions	Yuan et al., 2019
		Scaffolds	Improved cell attachment, proliferation, induction of cartilage formation	Lin et al., 2018
		Microspheres	Sustained Simvastatin delivery, induced proliferation of MC3T3-E1 cells, increased differentiation and bone mineralization	Terukina et al., 2016
		Microspheres	Sustained drug release, good biocompatibility	Nath et al., 2013
